# Emergency Medicine Scholarship in the Digital Age

**DOI:** 10.5811/westjem.2016.8.32283

**Published:** 2016-08-31

**Authors:** Mark I. Langdorf, Michelle Lin

**Affiliations:** * University of California, Irvine, Department of Emergency Medicine, Irvine, California; † University of California, San Francisco, Department of Emergency Medicine, San Francisco, California

How do scholars communicate in 2016 and beyond? Is the traditional journal being marginalized by digital communication? How many of us get the majority of our information from a paper page?

When an emergency medicine (EM) scholar has an idea, an innovation, or an important synthesis of existing information, is it appropriate to disseminate this several months later in a traditional print journal? In the digital age, when ideas flow around the world in minutes or hours, should scholarship flow so slowly? And yet, what is the role of peer review in attempting to assure that the ideas, innovations, and syntheses are generalizable, presented clearly, and (as best we can tell at the time) true?

What is the balance between digital communication and peer review?

As the editor of *WestJEM*, I consider this quandary frequently. The journal has grown substantially over the past eight years, with a current push circulation of 19,000 recipients and 4,500 paper copies per issue. Yet our digital exposure was 1.6 million pageviews and downloads last year, 12-fold greater than what we pushed to the academic world.

This switch from paper to digital information reminds me of the story of the buggy whip. As the world of 1920 changed from horse and buggy to the automobile, even the very best buggy whip maker in the world was doomed to go out of business. The transition took probably 30 years, but the outcome was inevitable. There would come a time when there was no need for buggy whips.

To cope with this transition, *West*JEM is partnering with the premier digital resource in academic EM, Academic Life in Emergency Medicine, or ALiEM. The journal embraces the concept that digital communication can and should be peer reviewed, and that the authors should get “traditional” academic credit proportional to their effort. This doesn’t mean that an educational blog post written over the weekend should get as much credit as a 5-year funded original research project published in a discriminating journal. Yet it deserves some credit, as the pace of innovation and dissemination accelerate.

Therefore we inaugurate *West*JEM: ALiEM “PROMPT”. PROMPT stands for Peer-Reviewed Online Media and Pedagogical approaches using Technologies. Although admittedly a mouthful, the title speaks to the immediacy of digital communication coupled with concepts of required peer-review and educational focus.

ALiEM (www.aliem.com) was originally a single-author educational blog focusing on clinical topics in emergency medicine (EM) in 2009. Since then it has evolved beyond a standard blog website into an innovative organization focusing on EM, health professions education, and digital scholarship. The organization’s efforts are focused on developing, sustaining, and growing four domains.

The first is the ALiEM blog itself, which garners an annual 1 million page views globally, and continues to publish such topics as clinical Tricks of the Trade, pocket Paucis Verbis cards, clinical reviews supplemented by expert peer reviews, book club discussions, and faculty development debates.

The second domain, in contrast to the blog creating a worldwide virtual community of practice, focuses on establishing smaller mentored communities through our Chief Resident, Fellowship, and Faculty Incubator models. As an example, our Chief Resident Incubator virtually mentors approximately 200 U.S. chief residents year-round.

The third domain is curriculum development. With so many different blogs and podcasts available in EM, our focus has partly shifted towards a more structured, curricular approach to education in the form of ALiEM University (ALiEMU, www.aliemu.com). This digital learning management system allows educators to track learner progress and to generate custom reports, while also allowing trainees to track their own personal learning progress. To date, existing ALiEMU e-courses include the CAPSULES series, which focuses on EM pharmacology; the Approved Instructional Resources (AIR) series, which is a monthly curated list of recent, high-quality blog posts and podcasts as vetted by an expert panel of EM educators; and the AIR-Professional series, which is a bi-monthly, expert-crowdsourced list of social media resources focusing on advanced clinical concepts.

And the fourth pillar is digital scholarship. With many academic educators and scholars on the ALiEM team, it was a natural evolution of our mission. Our team is comprised of a diverse, international group of primarily educator-scholars and academic leaders in medical institutions. Unlikely traditional organizations, the team also includes residents, medical students, and even pre-medical students, providing a diverse spectrum of ideas, perspectives, and skill sets. In total, we have over 100 volunteers who dedicate their time towards advancing health professions education and digital scholarship in the realm of EM and beyond. Collectively, the organization and its team members have published over 20 peer-reviewed publications about social media and digital scholarship in journals such as *Western Journal of Emergency Medicine*, *Annals of Emergency Medicine*, *Canadian Journal of Emergency Medicine*, *Journal of Graduate Medical Education*, *Academic Medicine*, and *Postgraduate Medical Journal*. Furthermore, the organization has partnered with various organizations and companies, such as the American College of Emergency Physicians, Council of EM Residency Directors, American Academy of Emergency Medicine, EBSCO Health, Call9, The Teaching Course, and AgileMD, to work on collaborative initiatives.

We, as readers, generators, and curators of academic scholarship, continue the journey toward digitalization together. I believe you will benefit from this unique cross-media partnership, and from the information published in these and subsequent issues of *WestJEM*’s ALiEM PROMPT.

